# The Potential of Graphene Nanoplatelets in the Development of Smart and Multifunctional Ecocomposites

**DOI:** 10.3390/polym12102189

**Published:** 2020-09-24

**Authors:** Pedro Pereira, Diana P. Ferreira, Joana C. Araújo, Armando Ferreira, Raul Fangueiro

**Affiliations:** 1 Centre for Textile Science and Technology (2C2T), University of Minho, 4710-057 Guimarães, Portugal; pedropereira@fibrenamics.com (P.P.); joanaaraujo@det.uminho.pt (J.C.A.); rfangueiro@dem.uminho.pt (R.F.); 2 Center of Physics, University of Minho, 4710-057 Braga, Portugal; armando.f@fisica.uminho.pt; 3 Department of Mechanical Engineering, University of Minho, 4710-057 Guimarães, Portugal

**Keywords:** GNPs, biodegradable polymers, natural fibers, piezoresistive behavior, multifunctional ecocomposite

## Abstract

Graphene and its derivatives have shown outstanding potential in many fields and textile/composites industry are not an exception. Giving their extraordinary properties, Graphene Nanoplatelets (GNPs) are excellent candidates for providing new functionalities to fibers and composites. In this work, natural fabrics (flax) were functionalized with chitosan (CS) based polymeric formulations of GNPs to develop fibrous systems with electrical properties as well as other functionalities. One of the greatest disadvantages of using carbon-based materials for fabrics’ impregnation is their difficult dispersion. Therefore, several polymers were used as matrices, binding and dispersive agents including chitosan, polyethylene glycol (PEG), and glycerol. All the systems were characterized using several techniques that demonstrated the presence and incorporation of the GNPs onto the composites. Besides their characterization, considering their use as smart materials for monitoring and sensing applications, electrical properties were also evaluated. The highest value obtained for electrical conductivity was 0.04 S m^−1^ using 2% of GNPs. Furthermore, piezoresistive behavior was observed with Gauge Factor (GF) of 1.89 using 0.5% GNPs. Additionally, UV (ultraviolet) protection ability and hydrophobicity were analyzed, confirming the multifunctional behavior of the developed systems extending their potential of application in several areas.

## 1. Introduction

The development of intelligent and multifunctional flexible fibrous systems is in full growth due to wide variety of potential applications, resulting from their flexibility and adaptability to different forms and shapes [1]. Smart fibrous structures with the ability to sense the environment/user can be used as sensors for electrocardiogram (ECG), electromyography (EMG), electroencephalography (EEG), movement, or even weight sensors [2]. Commercially, there are several examples of smart textiles containing hard cables and rigid electronic components that are not comfortable for the user, therefore, efforts should be made to use the fibrous structures themselves for the electronic functions. Thus, instead of attaching electronics to textile substrates, the fibrous structures can be functionalized with conductive materials to create electrically conductive surfaces and to form highly efficient conductive networks essential for the piezoresistive behavior of the materials (variation in resistance during compression) [3,4,5]. Piezoresistive sensors, which present simple circuit configuration, can be used to perform dynamic and static pressure measurements. To use fibrous systems as pressure sensors, the substrate must exhibit conductivity, and methods of providing conductivity to the textile have been widely studied. Materials like conductive polymers, metals, and metal oxide nanoparticles/nanowires, carbon based micron/nano materials, such as carbon particles, carbon nanotubes, carbon fibers and graphene, have been used and investigated [6,7].

Graphene is one of the most studied materials due to its exceptional physical properties including thermal conductivity and excellent mechanical and electrical performance. Pure graphene is not yet produced on a large scale and its price is still high. Thus, Graphene Nanoplatelets (GNPs) appear as an excellent alternative due to their low cost and the possibility of production on a large scale [8]. There are several examples in literature which exhibit the potential of GNPs for the development of smart fibrous structures with enhanced electrical properties. Nilsson et al. used a melt spinning technique to produce conductive textile fibers of polypropylene with hybridized GNPs [9]. Moriche et al. coated glass fiber fabrics with GNPs for strain monitoring applications [10]; flexible and wearable strain sensors were developed by Souri et al. using an ultrasonication technique for the coating of flax fabrics with GNPs [11]; flax fiber yarns and glass fiber yarns were coated by dip coating processes with GNPs by Mohan et al. [12], and Park et al. developed highly stretchable, sensitive, and wearable graphene strain sensors based on GNPs and poly(vinyl alcohol) yarns [13].

However, the fibers coating with GNPs and their effective dispersion into the fibers to obtain homogeneous and durable conductive surfaces is still a challenge. To overcome this issue, the use of polymeric matrices emerges as an alternative to improve the adhesion and dispersion degree of this type of carbon-based materials to the fibrous substrates allowing the development of GNP based composites. However, giving the growing interest in searching for new materials with improved environmental sustainability, attention is driven to ecocomposites based on the combined effect of natural fibers and natural or biodegradable polymers. Fibers like flax, jute or sisal have emerged as great alternatives due to their low cost, biodegradability, low weight, and abundance in nature [6,7,8,9,10,11,12,13,14]. Besides natural fibers, biodegradable polymers such as chitosan (CS) and polyethylene glycol (PEG) are also a target of interest due to their excellent characteristics such as biodegradability, non-toxicity, also being excellent dispersing agents for the GNPs [15,16].

Hence, the main goal of this work is the development of smart and multifunctional ecocomposites based on flax fabrics coated with optimized GNPs polymeric formulations composed by CS and PEG. Firstly, the flax fabrics were subjected to alkali treatment for cleaning the fabrics’ surface as well as to enhance the polymers’ adhesion to the fibers. After this, several GNP polymeric formulations using different GNPs percentages were optimized using CS and PEG as polymeric matrices and dispersive/binding agents as well as glycerol as plasticizer. The flax fabrics were further impregnated with the best formulation under study by the dip-pad-dry method. All the developed samples were properly characterized by Raman Spectroscopy, Field Emission Scanning Electron Microscopy (FESEM), and Thermogravimetric analysis (TGA). The electrical properties of the developed fibrous structures were evaluated including electrical conductivity, piezoresistive effect (the change in the resistance of materials caused by the structural deformations), and the sensitivity of the materials expressed as Gauge factor (GF) [17].

Finally, taking advantage of the multifunctional potential of graphene nanoplatelets, the UV protection ability as well as water repellency performance were evaluated increasing the potential of application of these smart ecocomposites as multifunctional materials. 

## 2. Materials and Methods 

### 2.1. Materials

CS (Acros Organics, Geel, Belgium) with molecular weight between 100,000–300,000 g mol^−1^, PEG (Sigma Aldrich, Germany) with molecular weight of 35,000 g mol^−1^ and the plasticizer glycerol (99%, Scharlab, Spain) were used as polymeric matrices. Flax fabrics were supplied by RCS (Braga, Portugal) produced using 100% flax natural yarns with 315 g m^−2^ of grammage. Sodium hydroxide (NaOH) (99%, Normax, Marinha Grande, Portugal) was used for the alkaline fiber pretreatment. As solvents, distilled water and acetic acid (99–100% p.a. Normax, Marinha Grande, Portugal) were used. The GNPs used were provided by Graphenest (Aveiro, Portugal), with 8–30 layers, thickness between 3 and 10 nm, and layers’ lateral dimensions of 0.5 to 0.2 μm and 150 m^−2^ g of surface area.

### 2.2. Sample Preparation

#### 2.2.1. Flax Fabrics Pretreatment

Flax fabrics were subjected to an alkaline pretreatment, to remove waxes and fats from its surface, to improve the adhesion between the polymers, the GNPs, and the fibers. To perform this treatment, an aqueous NaOH solution (1 M) was used. Subsequently, the fabric was placed in this solution for 60 min and stirred by orbital shaker. Finally, the fabrics were washed under running water to remove all residues and dried for 60 min at 80 °C.

#### 2.2.2. Optimization of the GNPs’ Polymeric Formulations

Initially, the polymeric formulations for GNPs dispersion were optimized for the flax fabric’s further impregnation. CS was used to improve the GNPs dispersion as well as to increase the GNPs adhesion to the fabric by the creation of possible anchoring sites between the cellulosic hydroxyl groups of flax and hydroxyl groups of CS. Besides CS, glycerol (Gly) was added as plasticizing agent to improve the fibrous systems malleability and PEG as a dispersive agent to facilitate the GNP’s dispersion.

In order to optimize the polymeric formulations, different percentages of CS, from 2 to 5% (*w*/*v*), Gly, 0.5 to 7.5% (*w*/*v*) as well as PEG, 2 and 5% (*w*/*v*) were tested and the best one obtained was used for the GNPs dispersion. Briefly, powdered CS was added to distilled water (2% (*w*/*v*)) and kept under magnetic stirring for 30 min using 350 rpm. After this time, 1% (*w*/*v*) of acetic acid was added slowly under magnetic stirring for more 30 min. After this, Gly was included (3% *w*/*v*) as well as PEG (2% *w*/*v*) and the solution was placed for 30 min in an ultrasound bath. 

Different percentages of GNPs were tested, from 0.1 to 2% (*w*/*v*). The GNPs were added to the previous optimized solution, mechanically stirred for 60 min and subjected to an ultrasound bath for 30 min. The fabrics’ impregnation was performed by the dip-pad-dry method, where 5 consecutive impregnations were carried out with a roller pressure of 60 Pascal, followed by drying in an oven at 80 °C for 2 h.

#### 2.2.3. Ground State Diffuse Reflectance and CIELAB Color Coordinates

The reflectance, R, from each sample was obtained with a Spectra Flash SF600 PLUS spectrophotometer supplied by Datacolor (Lucerne, Switzerland) in the spectral range from 360 to 700 nm. The remission function of fabrics, F(R), was calculated using the Kubelka–Munk equation for optically thick samples: F(R) = (1–R)^2^/(2R) = K/S, where K is the absorption coefficient and S the dispersion coefficient. Each sample was measured five times in different sites of the sample to ensure a relative homogeneity. CIELAB color coordinates were calculated with this Datacolor spectrophotometer using the difference Cielab coordinates D65/10 software (Lucerne, Switzerland) taking the untreated flax fabric as standard. The L* shows the lightness of the fabric; the tonality is expressed by the primary colors red, green, yellow and blue: +a* for red, −a* for green, +b* for yellow and −b* for blue. In this specific case, giving the graphene nanoplatelets’ dark color, special attention will be given to the lightness parameter. The lightness defines a gray scale between white and black. It is expressed by the variable L * and assumes 0 for absolute black and 100 for total white.

### 2.3. Samples Characterization 

#### 2.3.1. Thermogravimetric (TGA)

TGA analysis was used to obtain the maximum temperature at which a developed system resists without thermal degradation. The tests were performed on a STA 700 SCANSCI (Tokyo, Japan). The TGA trace was obtained in the range of 30 to 600 °C with a sweep rate of 20 °C min^−1^ under a constant flow of nitrogen (200 mL min^−1^). 

#### 2.3.2. Raman Spectroscopy 

The Raman Spectroscopy was used to analyze the structure and dispersion of GNPs into the fabrics, as well as the GNPs powder. Raman spectra were obtained on a Horiba LabRAM HR Evolution confocal microscope (Horiba Scientific, Longjumeau, France), equipped with a 532 nm (2.33 eV) laser. A 100× objective lens was used to focus the laser onto the sample. The samples, with an area of 40 × 20 mm^2^, were deposited into a glass slide. For each sample, an average of four scans was randomly collected, in order to ensure the analysis’ homogeneity. The results were analyzed using the LabSpec 6 software (also from Horiba Scientific, city, country).

#### 2.3.3. Field Emission Scanning Electron Microscopy (FESEM)

In order to study the impregnation and the degree of dispersion of the GNPs into the ecocomposites, FESEM analysis was used. The surface morphology and cross sections of the samples was analyzed by FESEM using the NOVA 200 Nano SEM equipment from FEI Company (Hillsboro, OR, USA). All samples were coated with a palladium-gold (Pd-Au) film (20 nm) to make them conductive.

### 2.4. Multifunctional Properties’ Evaluation

#### 2.4.1. Water Contact Angle Measurement (WCA)

To evaluate the hydrophobic character of the developed fibrous systems, WCA measurements were performed. For this, a contact angle system (dataphysics) coupled to a high-resolution camera was used. A volume of 5 μL of distilled water was dispensed from the syringe onto the fiber’s surface. For each sample, the contact angle was measured at 10 different locations, and the average and standard deviation for each test were calculated. It is important to note that, if the contact angle value is less than or greater than 90°, the sample is considered hydrophilic or hydrophobic, respectively. For values greater than 150°, the sample is considered superhydrophobic [1].

#### 2.4.2. UV Protection

The UV blocking properties of a fabric are evaluated by a UV protection factor (UPF), which is defined as the ratio of UV transmittance under the wavelength range of 280 to 400 nm, as the following equation shows:

(1) $$UPF=\;\frac{\int_{280}^{400}E_{\lambda }\times S_{\lambda }\times d_{\lambda }}{\int_{280}^{400}E_{\lambda }\times S_{\lambda }\times T_{\lambda }\times d_{\lambda }}$$

where $E_{\lambda }$ represents the relative erythemal spectral effectiveness, $S_{\lambda }$ the solar UV spectral irradiance, $d_{\lambda }$ the wavelength increment (nm), $T_{\lambda }$ the spectral transmittance of the specimen, and λ the wavelength (nm). The UPF, UVA, and UVB protection values of each sample were calculated according to EN13758-1. A Spectrophotometer UV-2600 (Shimadzu) with an ISR_2600 Plus detector was used and different places of the samples were analyzed, in order to assure the analysis’ homogeneity.

#### 2.4.3. Electrical Conductivity

For electrical conductivity tests, it was necessary to measure the electrical resistance of the material using an electrical source, which was connected to the material by conductive electrodes. By this method, it was possible to obtain the electrical resistance through the I–V curves (electric current intensity—voltage curves) [18]. The electrical resistivity is given by Equation (2):

(2) $$\rho =R\;\times \;\frac{A}{L}$$

where *ρ* represents the electrical resistivity (Ω m^−1^), *R* the electrical resistance (Ω), *A* the electrode area (mm^2^), and *L* the distance between electrodes (mm).

Electrical resistivity represents the ability of a given material to resist the flow of electric current. In other words, electrical conductivity is the inverse of electrical resistivity, as represented in Equation (3) [19]:

(3) $$\sigma =\;\frac{1}{\rho },$$

where *σ* represents the electrical conductivity (S m^−1^) and *ρ* the electrical resistivity (Ω m^−1^).

Electrical conductivity measurements were made using a Keitley 487 Picoammeter/Voltage Source by applying a potential difference between −1 to 1 V with a step of 0.1 V at room temperature. In order to be able to measure electrical resistance values, an electrode system with an electrode area of (5 × 1 mm^2^) and a distance between electrodes of 3 mm was developed. The electrical resistance was determined by the slope of the I–V curves, where Equation (2) was applied to obtain the electrical resistivity and then Equation (3), thus obtaining the electrical conductivity values.

**Figure 1:**
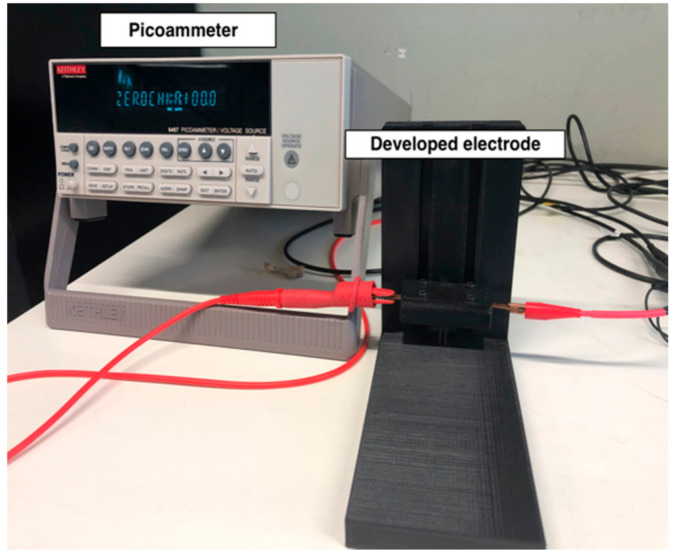
Equipment used for electrical resistance measurement.

Electrical resistance measurements were performed at three different points of the sample. Figure 1 shows the equipment used to measure electrical resistance.

#### 2.4.4. Piezoresistive Tests

**Figure 2:**
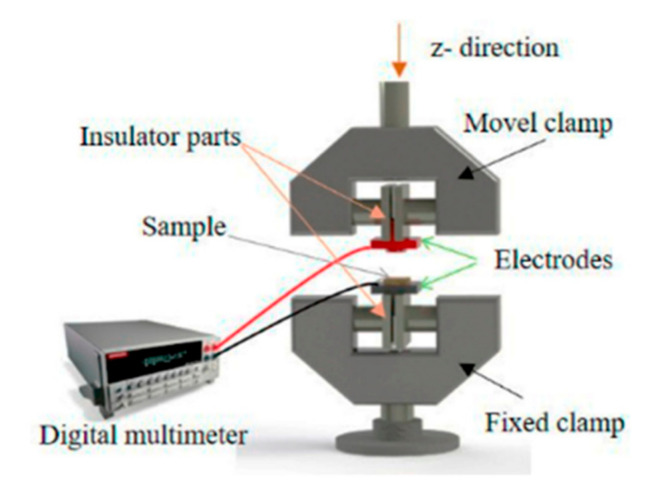
Schematic representation of the experimental configuration of the clamps for the compression experiments with simultaneous electrical measurements for electro-mechanical response evaluation of the samples.

For the piezoresistive tests, two separate devices were used simultaneously: a Shimadzu-AG-IS universal testing machine with a 1000 N load cell, with a *Z*-axis deformation of 0.5 mm and a compression speed of 4 mm min^−1^ in the sample with a diameter of 10 mm during 10 cycles. To measure the variation of electrical resistance, two electrodes were placed in the clamps, as can be observed in the diagram of Figure 2. Through these electrodes connected to a Keithley 2700 digital multimeter, it was possible to record the electrical resistance variation over 10 compression cycles. 

In order to quantify the piezoresistive effect, it was necessary to calculate the mechanical deformation (*ε*) and the electrical resistance variation (ΔR). For the calculation of *ε*, Equation (4) was used:

(4) $$\varepsilon =\;\frac{z}{d},$$

where z represents the vertical displacement (mm), and d the initial sample thickness (mm). The gauge factor (GF) is the parameter that quantifies the sensitivity of a piezoresistive sensor. This parameter consists of the variation of the electrical resistance per unit of mechanical deformation, as represented in Equation (5) [20]:

(5) $$GF=\;\frac{∆R/R_{0}}{\varepsilon },$$

where *ΔR* is the change in resistance caused by deformation, and *R*_0_ the resistance before deformation. *ε* is the mechanical (dimensionless) strain [21,22].

For the calculation of GF, the curves of ΔR as a function of **ε** were plotted for the ten upward cycles of each test. Through a linear regression, the slope of each one of them was determined, and this slope corresponds to the GF of the samples.

## 3. Results

As referred to before, the main goal of this work was the development of smart and multifunctional ecocomposites based on the combined effect of flax fabrics, GNPs, Chitosan, and PEG. For this purpose, several polymeric solutions, composed by CS, PEG, and Gly, were optimized, and also the concentrations of GNPs added to the polymeric formulation (from 0.1 to 2%). 

**Figure 3:**
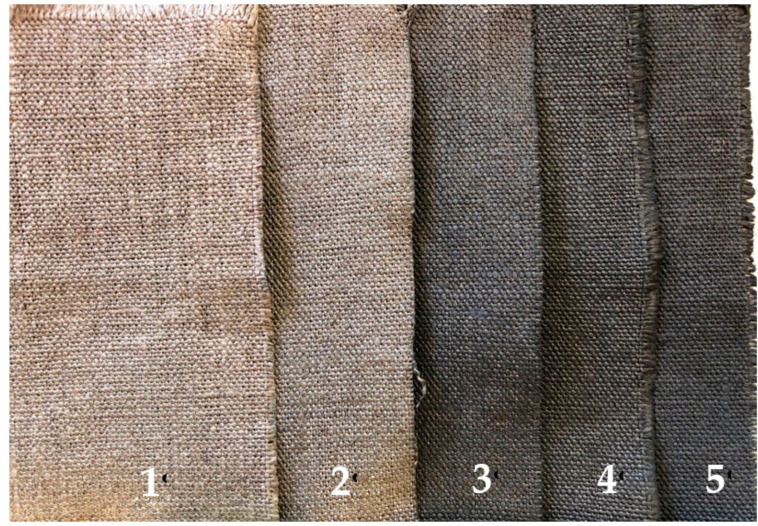
Images of the developed samples: (1) FlaxCPG, (2) FlaxCPG + 0.1%, (3) FlaxCPG + 0.5%, (4) FlaxCPG + 1% and (5) FlaxCPG + 2%

Figure 3 exhibits the flax fabrics impregnated with these formulations without GNPs (FlaxCPG) for comparison purposes and the flax fabrics impregnated with different formulations of CS, PEG, and Gly using several percentages of GNPs: 0.1% (FlaxCPG0.1%), 0.5% (FlaxCPG0.5%), 1% (FlaxCPG1%), and 2% (FlaxCPG2%).

Regarding Figure 3, it is possible to observe that the developed samples exhibited a homogeneous coating due to the color consistency all over the fabric. In addition, with increasing percentages of GNPs, the samples become darker as expected. Cielab lightness parameters presented in Table 1 were acquired in five different sites of each sample to evaluate the homogeneity of the GNPs’ impregnation. At the same time, comparing the L* mean values of each sample, it is possible to infer about the samples’ color change. With increasing percentages of GNPs formulation (0.1, 0.5, 1, and 2%), the L* value when compared with the flax fabric value decreased from 52.2 to 33.9, indicating that the samples become darker with higher contents of GNPs. In addition, the color gradually becomes darker as the amount of GNPs increase. As referred to before, L* were measured in five different places of each sample under study and the mean and standard deviation of the obtained values for the color coordinate were calculated. Overall, the samples’ color was homogenous all over the fabrics’ surface, since the calculated standard deviation values for L* coordinate and for different places of the sample were very low.

In our opinion, the use of natural fibers and polymers with hydroxyl groups promoted the homogeneous dispersion of the GNPs onto the fabrics as well as their strong anchoring to the fibers. Due to the presence of the hydroxyl groups of cellulose (from the flax), it was expected the formation of hydrogen bonds between cellulose and the PEG groups, which ultimately improved the incorporation of the GNPs onto the flax fabric. Analogously, the use of CS also contributed for the adhesion between the GNPs and flax fabric conferring a more rigid behavior to the fabric. On the other hand, the addition of Gly was essential to instill some flexibility to the ecocomposite (restricted by the addition of CS), which is a very important parameter for the final application of these developed systems as flexible sensors. Given the pressure applied during the dip-pad-dry impregnation process, it was possible to remove the excess of agglomerates particles that the fabrics could not adsorb. Otherwise, this excess of agglomerates would remain on the fabric surface turning its dyeing inhomogeneous.

Overall, the samples exhibited an effective functionalization with the GNPs polymeric formulations as it will also be analyzed in the following sections by TGA and Raman spectroscopy.

### 3.1. TGA

**Figure 4:**
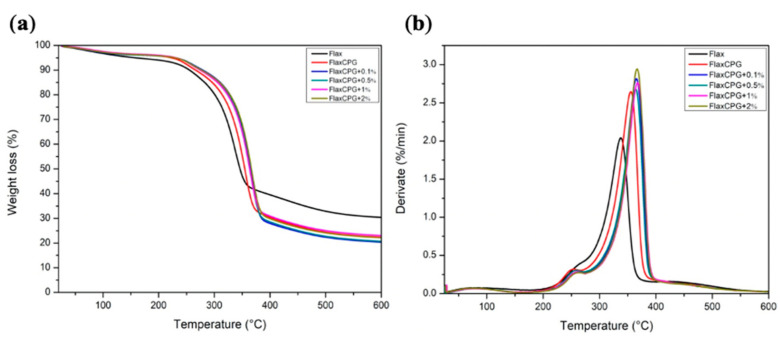
TGA (**a**) and DTG (**b**) curves of Flax, FlaxCPG and FlaxCPG with different percentages of GNPs.

TGA analysis was performed in order to evaluate the effect of GNPs addition on thermal stability of the fibrous systems. The TGA curves with the corresponding derivatives (DTG) are shown in Figure 4.

In Figure 4, all the samples showed an initial weight loss, up to approximately 100 °C, as a result of water evaporation. The second step, around 253 °C in all spectra, is related with hemicellulose degradation from the flax fibers [23]. The maximum degradation peaks of CS and PEO normally appear between 300 °C and 400 °C as one decomposition step [24,25]. Their presence in these spectra are not so pronounced due to superposition with the region corresponding to degradation of hemicellulose and cellulose from flax fabric, which are in a higher amount in the samples than either chitosan or PEG. For the flax sample, at 337 ºC, the crystalline cellulose degradation occurs; however, with an increasing percentage of GNPs onto the ecocomposite, the degradation temperature increases to 367 °C (2% of GNPs). The incorporation of GNPs increased the thermal stability of the system in 30°C allowing the development of a more thermally stable system essential for electronic applications [26]. Furthermore, the fabrics’ alkaline pretreatment removed some constituents on its surface (namely possible fats); however, the lignin degradation step remains between 400 and 500 °C although not very pronounced [27].

### 3.2. Raman Spectroscopy 

**Figure 5:**
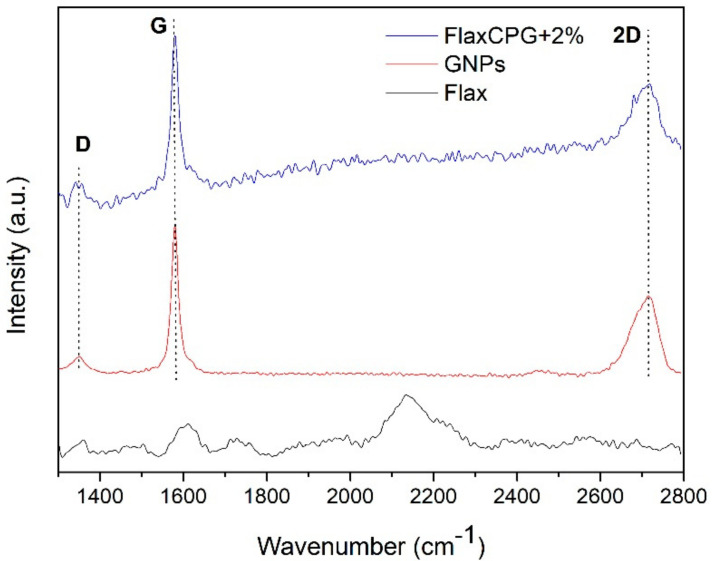
Raman spectra of the flax fabric (black), GNPs powder (red) and the developed FlaxCPG + 2% sample (blue).

Raman spectroscopy is known as the best technique for carbon-based materials’ structural characterization and detection, providing valuable information about defects and stacking of the GNPs. Raman Spectroscopy tests were performed to study the structure of the GNPs used in this work and to verify their presence in the developed samples. Figure 5 shows the spectra of GNPs powder used in this work in comparison with the developed sample using 2% of GNPs formulation (FlaxCPG + 2%).

The Raman spectra of Figure 5 exhibits the presence of three characteristic bands of carbon-based materials, which correspond to the band D, G, and 2D band. The 2D band form, representative of graphite, provides information about the GNPs number of sheets. Another method of analyzing the number of GNPs sheets is by calculating the I2D/IG ratio. Band D gives information about the presence of sp^3^ hybridized carbon, i.e., it gives information on the level of defects present in the structure [23]. The G band gives information about the plane vibrations of the sp^2^ hybridized carbon atoms. Through the relationship of bands D and G (ID/IG), it is possible to obtain information on the structure, size, and defects of the carbon material [26,28].

The GNP’s powder spectrum shows the D band peaking at approximately 1350 cm^−1^ with relatively low intensity when compared with G band, which indicates that these GNPs have few structural defects, presenting an ID/IG of 0.08. The G band with higher intensity appears at 1580 cm^−1^ and 2D band at ~2720 cm^−1^, with a lower intensity than the G band (I2D/IG = 0.52), indicating that this material is composed by graphene multilayers. As well as flax sample, the ecocomposite spectrum also exhibits the characteristic bands of GNPs, indicating their presence into the composite surface. The band intensities are lower when compared to the GNP’s powder spectrum because the fabric does not have a completely flat surface, which influences the acquisition sensitivity of Raman signal.

### 3.3. Field Emission Scanning Electron Microscopy (FESEM)

**Figure 6:**
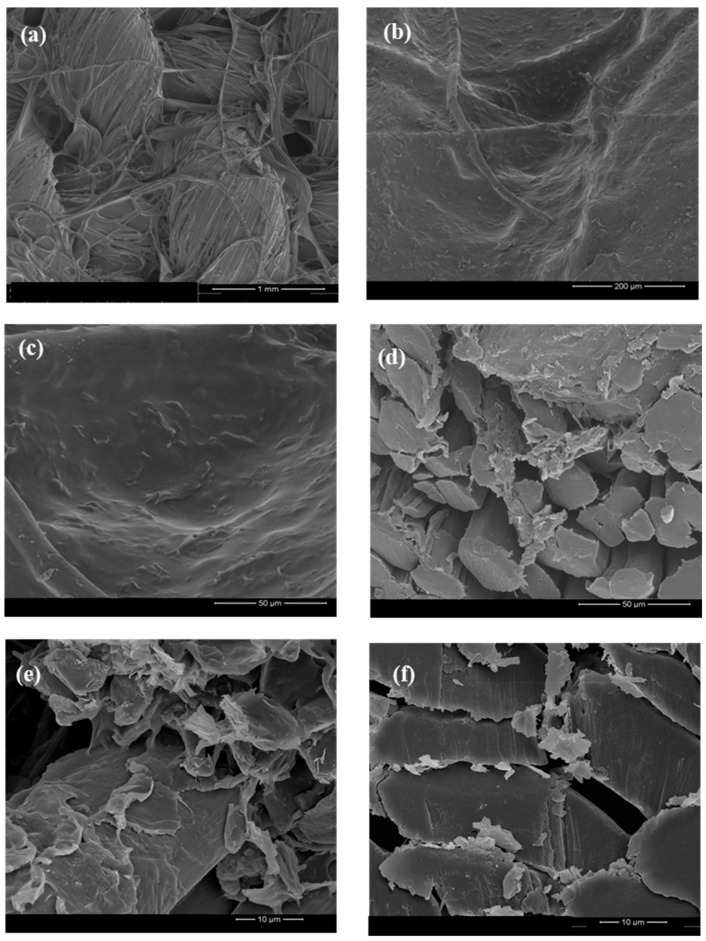
FESEM images of the ecocomposites surface with 2% of GNPs using different magnifications: (**a**) 1 mm, (**b**) 200 μm, and (**c**) 50 μm and cross section images using 50 μm (**d**) and 10 μm (**e**) and (**f**) in different zones of the sample.

The FESEM technique was used to infer about the distribution of the GNPs onto the ecocomposites as well as to analyze the surface coating based on the polymeric matrices under use. Images from the ecocomposites’ surface (Figure 6a–c) and samples’ cross section (Figure 6c–e) were obtained with different magnifications taking as example the sample with 2% of GNPs. 

Figure 6a–c show the distribution of the GNPs along the fabric and the clear coating of the fibers with the polymers. Furthermore, the presence of the GNPs under the CS/PEG/Glycerol coating is possible.

In Figure 6c–e, it is possible to observe the cross-section images of the sample FlaxCPG + 2%. These three images show the distribution of the GNPs between the fibers of the flax fabric. Moreover, it is also possible to visualize with more detail in Figure 6e the deposition of GNPs on the fiber surface. In general, through these images, it is possible to conclude that the coating is effective without presenting problems related to the weak adhesion between the polymeric matrix and the fibers. In addition, the polymeric coating formed around the GNPs seems to favor the anchoring of these nanoparticles to the fiber surface.

### 3.4. Electrical Properties 

The electrical properties depend on certain characteristics, such as GNPs quantity, dispersion, agglomeration, and conductive pathways formation in the substrate [29]. In order to study the influence of the GNPs percentage on the electrical conductivity, the electrical resistance was measured in three different points of the sample, obtaining an average value. Table 2 shows the electrical conductivity values calculated as referred before in the experimental part for the several samples under study using the two-point probe method.

**Figure 7:**
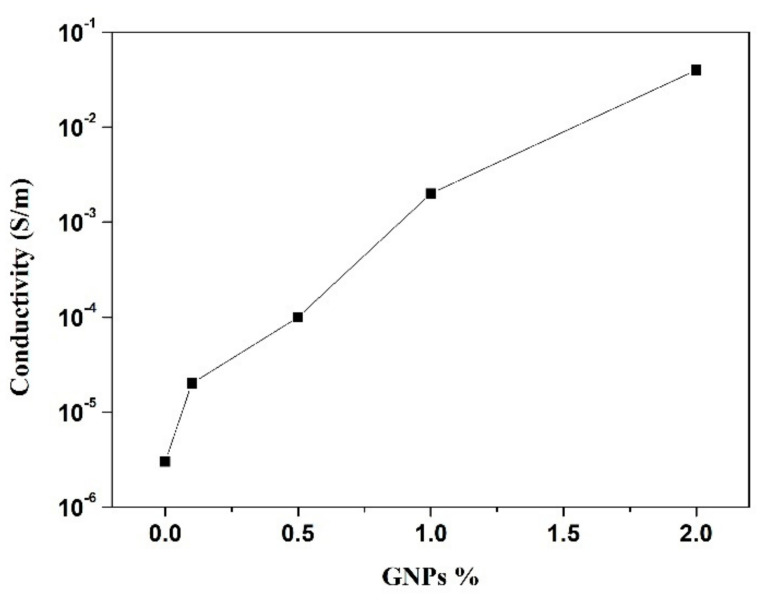
Graphic exhibiting the dependence of conductivity values with GNP percentage.

As shown in Table 2 and Figure 7, the introduction of GNPs increased the values of electrical conductivity, proving the electrical properties of these NPs and validating what is reported in several studies about their excellent electrical conductivity [30,31]. 

**Figure 8:**
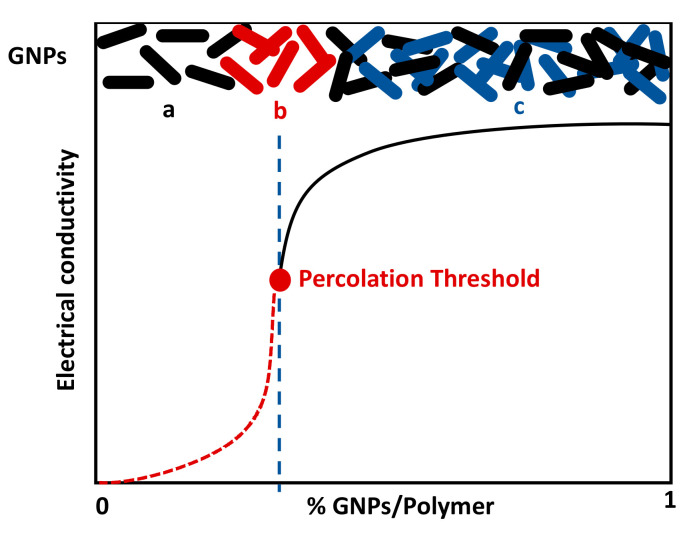
Percolation threshold of composites—adapted from [32].

For small percentages (below 0.5% GNPs), the values of electrical conductivity are very low; however, with 2%, the value reaches 0.04 S m^−^^1^. In the literature, the percolation threshold theory is presented as the range where the electrical conductivity of the samples varies by some orders of magnitude within this limit and where there is a very small variation in conductivity before and after that limit [19]. In Figure 8, the percolation threshold is represented, and it is observed that the variation in the conductivity of GNPs/polymer composites can be divided into three phases.

In the first phase (zone a), the electrical conductivity is very low due to the low percentage of GNPs. On the other hand, with the increase of the GNP percentage, there is a formation of large interconnected clusters of these NPs (zone b). Thus, there is a huge increase in electrical conductivity values. This phase is very important to obtain the best piezoresistive response. Finally, in the third region (zone c) with the increase of the GNPs percentage, there’s a small increase in conductivity that becomes stable after the addition of a certain amount of GNPs [32,33].

When we compare the values of electrical conductivity obtained experimentally with the percentage of GNPs used, only the first two phases are verified. In addition, 4% of GNPs was tested in order to reach the plateau; however, due to the high quantity of GNPs, their dispersion into the polymeric formulation became very difficult, and the final ecocomposite presented a very heterogeneous coating. Due to this heterogeneous surface, the conductivity values obtained in several sites of the sample exhibited a very high associated error.

Taking into account the main objective of this work, the development of a piezoresistive fibrous system, the electrical response under mechanical compression of the developed samples was evaluated. It is shown that, when measuring the electromechanical properties in a nanocomposite with percentages of GNPs/Polymer near the percolation threshold, there will be a greater sensitivity to mechanical deformation which in turn will cause greater variations in electrical resistance in the nanocomposites, which means higher GF values [34]. Far from the percolation threshold, electrical resistance variations are smaller and therefore their effect in the electrical response is also small [35].

**Figure 9:**
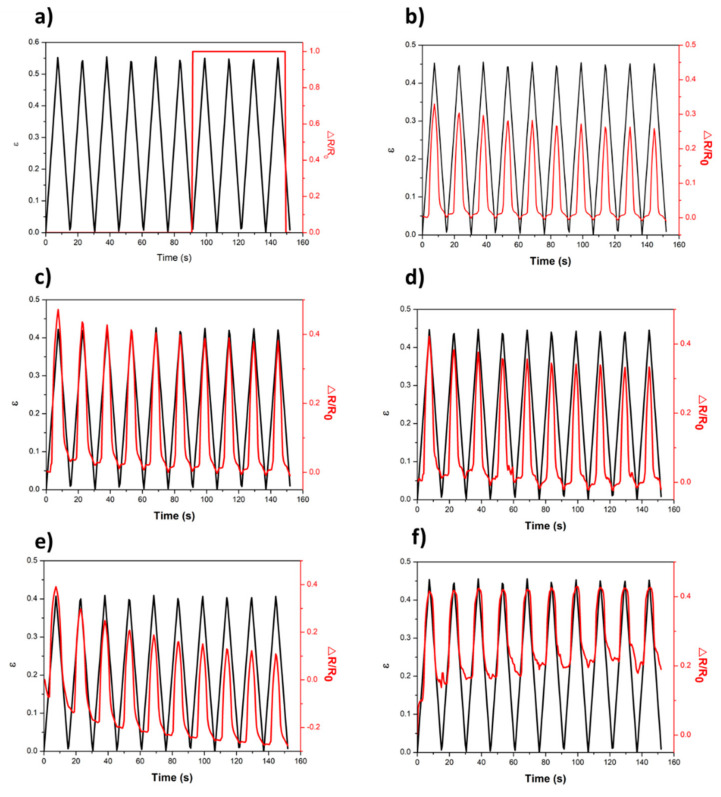
Piezoresistive response of flax fabric with different percentages of GNPs. (**a**) Flax, (**b**) FlaxCPG, (**c**) FlaxCPG + 0.1%, (**d**) FlaxCPG + 0.5%, (**e**) FlaxCPG + 1%, and (**f**) FlaxCPG + 2%.

Firstly, only the fabric was analyzed and, as expected, no piezoresistive behavior was observed (Figure 9a). The change in electrical resistance values presented at approximately 90 s is related with fabric “fatigue” that does not recover to its initial shape over the several applied cycles. The pressure applied over several cycles induces the separation between the fibers of the fabric, creating empty spaces between them. Thus, the electrodes come into contact short circuited presenting electrical resistance values that are not real. In all of the other cases (from b to f), the electrical resistance changes with the applied strain, and this tendency is maintained for the 10 cycles, for FlaxCPG samples with different percentages of GNP (0.1 to 2%).

**Figure 10:**
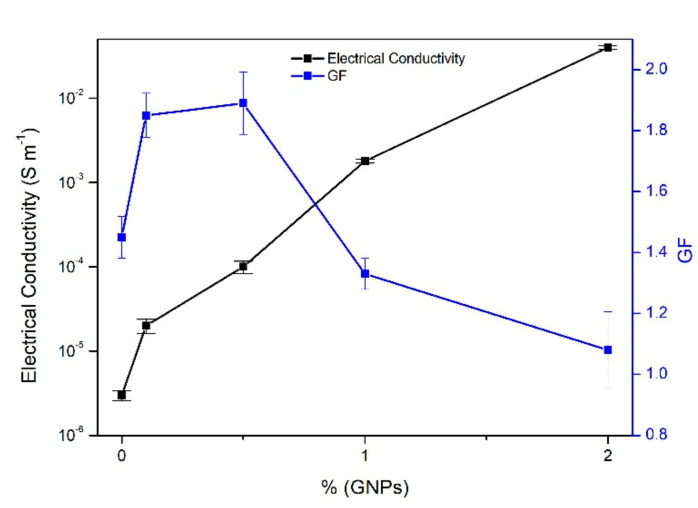
Relation between electrical conductivity and GF.

It can also be observed that the electrical resistance changed linearly with the applied strain and was maintained for the different cycles and for the different samples. For the FlaxCPG + 1% sample, Figure 9e, there is a decrease in response over the cycles, and this behavior could be attributed to fabric fatigue, which is unable to return to its initial state. The sample with 2% of GNPs, Figure 9f), only presents electrical response for ranges of strain between approximately 0.15 and 0.4, which demonstrates lower sensitivity for low pressures. Gauge Factor values where calculated for the five different samples using Equation (4). The slope of the linear fit (obtained with a R-square of 0.97) corresponds to the GF of each sample presented in Table 3. These GF values where calculated in each ascending cycle of Figure 9 and the values are the average of the ten ascending cycles. Figure 10 shows the relation between the electrical conductivity and the calculated gauge factors for each sample under study.

With the analysis of GF values, it was confirmed that 0.5% of the GNP system displays the best performance, which is near the percolation threshold. For the percentage of 1% of GNPs, a reduction in the GF values was verified. 

From Figure 10, for percentages between 0.1 and 0.5% of GNPs, there is an increase in both properties, conductivity, and GF. From the percentage of 0.5% of the GNPs and up to 2% of the GNPs, the trend is the decrease of GF and the increase of the electrical conductivity, so the higher the value of the electrical conductivity, the lower the value of GF. According to these results, the percentages between 0.5 and 1% of GNPs (with higher GF) are the best systems to be used as piezoresistive elements in sensors’ applications. 

### 3.5. Multifunctional Properties: UV Protection and Water Repellency Behavior

**Figure 11:**
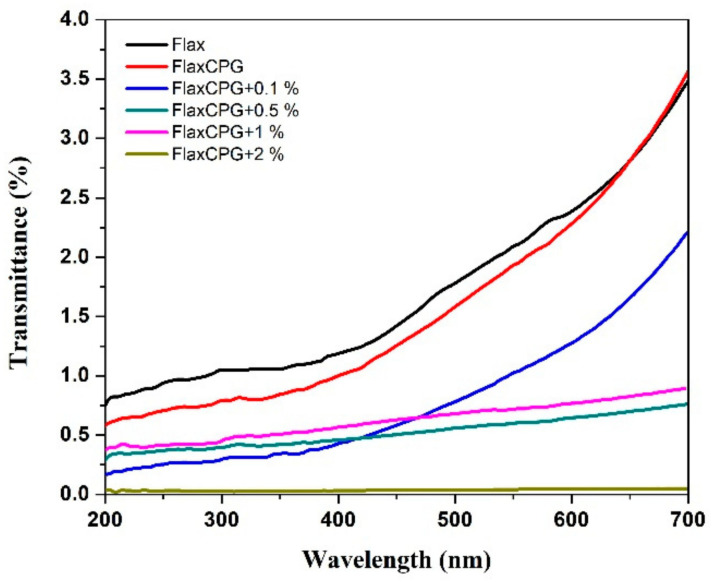
Transmittance spectra of the several samples under study.

Besides the electrical properties and with the purpose of evaluating the potential of these developed systems as multifunctional structures, the UV protection ability and water repellency behavior (hydrophobicity) was studied. Figure 11 exhibits the transmittance spectra obtained for all the samples under study.

The UV transmittance of the flax fabrics with and without the developed coatings with different percentages of GNPs was evaluated, as well as their UV protection behavior. The transmittance spectra clearly showed a difference between the transmittance values of the uncoated sample when compared with the coated ones. As expected, this distinction was more accentuated specially for the flax fabrics coated with the formulations containing GNPs when compared with the sample coated only with the polymeric formulation (without GNPs).

The samples with GNPs presented very low transmittance percentages, especially between 200 and 400 nm. These results imply that the addition of a GNPs’ coating to the flax fabrics can effectively enhance the UV protection of this material.

An efficient protection of the sun’s malicious effects needs to have components that absorb both UVA and UVB radiation before it reaches the skin. Thus, the development of materials that present this characteristic is of great importance [36].

The rate of UV protection was assessed by the calculation of UPF values, and the percentages of UVA and UVB blocking were also calculated. The uncoated flax fabrics already presented a good UPF value (95), which represents a classification of excellent (50+). However, with the addition of 1 % of GNPs into the coated samples, the UPF value increased to 211, which means that less UV rays can penetrate the samples, when compared with the uncoated ones. An increase in the UVA and UVB blocking percentages was also verified. For the flax fabrics, the obtained UVA and UVB protection values were 98.91 and 98.95%, respectively. For the sample coated with the formulation with 1% of GNPs, the UVA and UVB percentages increased to 99.48 and 99.52, respectively, reaching almost 100%. It is possible to affirm that GNPs can effectively block UV rays, which is in accordance with literature. Qu et al. demonstrated that the functionalization of cotton fabrics with GNPs improved the protection against UV radiation of the fabrics, with an increase of the UPF values from 32.71 to 356.74, with the addition of GNPs. They also concluded that the UV blocking offered by these types of particles could be due to the combination of the absorption effect of the GNPs for wavelengths shorter than 281 nm and the reflection at wavelengths higher than this value, which results from the unique 2D planar structure of graphene [37]. Hu et al. also demonstrated the UV protection capability of graphene. They coated cotton fabrics with a formulation of graphene/polyurethane, and improved the UPF values of the pristine cotton fabrics from 8.91 to 500 for the fabric with 0.8-wt% graphene [38].

Despite UV protection, the water repellency property was analyzed for the developed ecocomposites. Textile substrates with hydrophobic capabilities have received a lot of attention due to their liquid repellency, self-cleaning, unidirectional liquid transport, and barrier coating on fiber surfaces [39].

**Figure 12:**
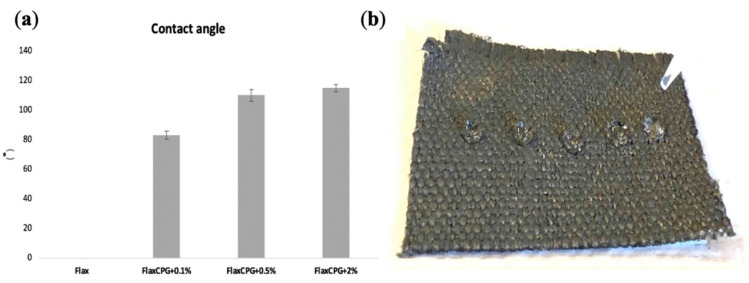
(**a**) average WCA obtained for treated flax, FlaxCPG + 0.1%, FlaxCPG + 0.5%, FlaxCPG + 2% and (**b**) WCA test of sample with 2% GNPs.

To study the hydrophobicity of the developed fibrous systems, they were subjected to WCA tests. For each sample, 10 tests were carried out, in different locations. Figure 12a shows the average water contact angle obtained for treated flax, FlaxCPG + 0.1%, FlaxCPG + 0.5%, FlaxCPG + 2% b and WCA test of sample with 2% GNPs.

As shown in Figure 12a, treated flax has a contact angle of approximately 0°. This result was already expected due to the alkaline treatment carried out on the fabric, which led to the removal of several impurities such as waxes, fats, and some lignin [40]. 

With the addition of GNPs, the WCA increases; however, with 0.1% of GNPs, the fabrics still present a hydrophilic nature (WCA was around 83° ± 2.53°). For the remaining percentages (0.5 and 2 % GNPs), there was an increase in the water contact angle to 110° ± 3.90° and 115° ± 2.37°, respectively, which means that GNPs create a totally hydrophobic surface. It’s possible to affirm that we started with a hydrophilic surface, the uncoated flax, and, with the addition of GNP percentages higher than 0.1 %, we obtained hydrophobic surfaces, reaching a contact angle of 115° for the sample with the higher concentration. These results are in concordance with previous studies that confirm the influence of GNPs to produce hydrophobic structures, making it capable to be used for several applications [39,41]. For example, Prolongo et al. studied the influence of the addition of GNPs in the hydrophobicity of epoxy resin composites. They concluded that the addition of GNPs led to a significant increase of the hydrophobicity of the composites, since the epoxy resin by itself presented a contact angle of 70º and the GNP/epoxy composites presented a contact angle in the range of 92–104° [42].

## 4. Conclusions

The main goal of this work was the development of a piezoresistive and multifunctional ecocomposite based on the combined effect of GNPs, flax fabrics, and biopolymers like CS and PEG. FESEM, Raman spectroscopy, and ATR-FTIR analysis validated the presence of GNPs onto the developed systems and confirmed the flax fabrics coating with biopolymers. Moreover, the successful impregnation by the dip-pad-dry method was also visible giving the homogeneous distribution of the GNPs/biopolymers formulation all over the fibrous substrates. Besides these characterization methods, thermogravimetric analysis was performed for all the samples under study, and it was shown that the thermal stability of the developed systems increased with the use of higher GNP percentages.

Simultaneously to the characterization, the electrical properties such as conductivity and piezoresistive response were evaluated. A maximum electrical conductivity value of 4.0 × 10^−2^ S m^−1^ was obtained using the polymeric formulation with 2% of GNPs. Regarding the piezoresistive tests, the electrical resistance changed linearly with the applied strain and was maintained along the pressure cycles under study. The best values of gauge factor were obtained for the ecocomposite with 0.5% of GNPs.

The multifunctionality of the developed fibrous systems was also evaluated and validated. Regarding hydrophobicity, the addition of GNPs improved the hydrophobic properties, reaching values of water contact angle at nearly 115° corresponding to hydrophobic behavior (for the system with 2% of GNPs). Moreover, the ecocomposites also exhibited UV protection capacity with UPF values of 50+.

In summary, we proposed a simple, scalable, and cost-effective method for developing piezoresistive sensors based on conductive flax ecocomposites. These systems can be used for pressure sensing related applications and detecting relatively high loadings, such as, for example, weight differences and human motions. In addition to their electrical properties, high UPF values and hydrophobic behavior can be of great importance, considering their application as possible flexible fibrous substrates with self-cleaning and UV protection ability. 

## Figures and Tables

**Table 1:** Cielab lightness coordinates measured in five different sites of each sample under study.

	Flax	FlaxCPG	FlaxCPG + 0.1%	FlaxCPG + 0.5%	FlaxCPG + 1%	FlaxCPG + 2%
Lightness parameter	L*	L*	L*	L*	L*	L*
Measurement 1	52.1	51.69	48.53	36.35	37.77	34.17
Measurement 2	51.7	50.95	48.95	37.36	37.27	34.97
Measurement 3	50.23	43.74	53.95	38.86	36.87	33.19
Measurement 4	53.27	48.98	54.58	36.83	36.95	32.98
Measurement 5	53.66	52.39	42.68	36.56	35.74	34.68
Mean	52.2	49.7	49.6	37.2	36.9	33.9
Standard deviation	1.2	3.1	4.3	0.9	0.7	0.8

**Table 2:** Electrical conductivity values of the developed fibrous systems and corresponding error.

Samples	Electrical Conductivity (S m^−1^)	Error
Flax	-	-
FlaxCPG	3.0 × 10^−6^	±4.0 × 10^−^^7^
FlaxCPG + 0.1%	2.0 × 10^−5^	±3.8 × 10^−^^6^
FlaxCPG + 0.5%	1.0 × 10^−4^	±1.7 × 10^−^^5^
FlaxCPG + 1%	1.8 × 10^−3^	±1.0 × 10^−^^4^
FlaxCPG + 2%	4.0 × 10^−2^	±2.0 × 10^−^^3^

**Table 3:** Average GF values obtained for each sample.

Samples	GF	Error
FlaxCPG	1.45	0.06
FlaxCPG + 0.1%	1.85	0.07
FlaxCPG + 0.5%	1.89	0.10
FlaxCPG + 1%	1.33	0.05
FlaxCPG + 2%	1.08	0.12
